# Polysaccharides As Viscosupplementation Agents: Structural Molecular Characteristics but Not Rheology Appear Crucial to the Therapeutic Response

**DOI:** 10.3389/fmed.2017.00082

**Published:** 2017-06-19

**Authors:** Rita C. Machado, Susana Capela, Francisco A. C. Rocha

**Affiliations:** ^1^Rheumatology and Metabolic Bone Diseases Department, Hospital de Santa Maria, CHLN, Lisbon Academic Medical Centre, Lisbon, Portugal; ^2^Rheumatology Research Unit, Faculty of Medicine, Instituto de Medicina Molecular, University of Lisbon, Lisbon, Portugal; ^3^Faculty of Medicine, Department of Internal Medicine, Federal University of Ceará, Fortaleza, Brazil

**Keywords:** viscosupplementation, hyaluronic acid, polysaccharide, osteoarthritis, pain, cartilage, guar gum

## Abstract

**Introduction:**

Most clinical studies and basic research document viscosupplementation (VS) in terms of effectiveness and safety, but only a few highlight its molecular mechanisms of action. Besides, there is generally focus on hyaluronic acid (HA) as being the most relevant polysaccharide to reach the clinical endpoints, attributing its effect mainly to its unique viscoelastic properties, related to a high-molecular weight and gel formulation. Usually, studies do not approach the possible biological pathways where HA may interfere, and there is a lack of reports on other biocompatible polysaccharides that could be of use in VS.

**Aim:**

We briefly review the main proposed mechanisms of action of intra-articular hyaluronic acid (IA-HA) treatment and discuss its effectiveness focusing on the role of rheological and intrinsic structural molecular properties of polysaccharides in providing a therapeutic effect.

**Methods:**

We conducted a literature search using PubMed database to find articles dealing with the mechanisms of action of IA-HA treatment and/or emphasizing how the structural properties of the polysaccharide used influenced the clinical outcomes.

**Discussion/conclusion:**

HA is involved in numerous biochemical interactions that may explain the clinical benefits of VS, most of them resulting from HA–cluster of differentiation 44 receptor interaction. There are other important aspects apart from the molecular size or the colloidal state of the IA-HA involved in VS efficiency that still need to be consolidated. Indeed, it seems that clinical response may be dependent on the intrinsic properties of the polysaccharide, regardless of being HA, rather than to rheology, posing some controversy to previous beliefs.

## Introduction

Hyaluronic acid (HA) is a non-sulfated polyanionic polysaccharide that can be highly viscous, formed by synoviocytes, fibroblasts, and chondrocytes inside joints. It is present in the synovial fluid and the extracellular matrix of the cartilage (1) where it naturally occurs as a high-molecular weight (HMW) substance with an average molecular weight (MW) of 6 × 10^6^ Da (2).

In healthy joints, HA is thought to act mainly as a viscoelastic shock absorber during high shear and as a lubricant during slow movement (3), apparently due to its rheological properties. There are claims that reduction of the MW and concentration of HA that occurs in osteoarthritis (OA) would alter the rheological properties of HA resulting in increased friction and reduced protection of the articular cartilage during mechanical stress (2, 4). Viscosupplementation (VS)—the injection of exogenous HA into the synovial joints—has emerged as a therapeutic approach to restore the viscoelasticity of the synovial fluid in the joint (2).

The observation that the clinical relief after a single injection of this compound may last up to 12 months (5, 6) argues against a pure rheological effect to explain the analgesia considering that exogenous HA possibly lasts less than 1 day in the joint (5). The possibility that intra-articular hyaluronic acid (IA-HA) has disease modifying properties does also question a mechanical action as the only explanation for the therapeutic benefit. Several papers have emphasized other clinical benefits arising from VS, such as chondroprotection and induction of proteoglycan and glycosaminoglycan synthesis, as well as anti-inflammatory, subchondral bone sparing, and analgesic actions. Altman et al. (7) summarized the mechanisms of action of VS of the knee described in the literature in a systematic review, with HA–CD44 receptor binding being the most frequently reported source of the effects mentioned.

According to some reports, the therapeutic efficacy of hylans (HA derivatives) is directly associated with their viscoelastic properties, related to their HMW and gel formulation, despite a lack of studies to prove this assumption (5, 8).

Indeed, the clinical benefit resulting from HA preparations injected into OA joints was also obtained with preparations of lower MW (in the order of 1 × 10^6^ Da or less) (9). Further, the benefit of using a viscous or saline-soluble compound has not been brought to scrutiny. In keeping with this statement, it was shown (8) that the administration of a polysaccharide derived from guar gum (GG) injected into the knee of rats subjected to anterior cruciate ligament transection (ACLT), used as an OA model, provided significant analgesia that was similar to Hylan G-F 20™, as a comparator, regardless of using a saline-soluble or a reticulated, highly viscous, polysaccharide preparation, this way suggesting that polysaccharide compounds used in VS provide analgesia independent of the gel state (8).

The controversy on whether HA preparations are worth in treating OA persists. Whereas a recent systematic review and meta-analysis considered IA-HA effective for pain relief in knee OA (10), this therapy was ruled out by the American Academy of Orthopedic Surgeons OA guidelines (11). We do not intend to discuss the controversy on the clinical efficacy or relevance of using “viscosupplements” in OA treatment. Rather, we briefly review some of the proposed mechanisms of action of VS while strictly addressing the issue that the intrinsic polysaccharide structure, rather than its rheological properties, probably account for the pharmacologic effect linked to the analgesia provided by this therapy.

## Mechanisms of Action

The most relevant clinical results of VS described are chondroprotection, induction of proteoglycan and glycosaminoglycan synthesis, mechanical, anti-inflammatory, subchondral, and analgesic actions (7). Mechanisms of action refer to the specific biochemical interaction through which HA as a molecule produces the listed pharmacological effects. Most of them result from the interaction between HA and various cellular receptors such as the CD44 receptor, the intercellular adhesion molecule (ICAM)-1, and the receptor for hyaluronan-mediated motility (RHAMM).

### The Role of CD44 Receptor

CD44 is a cell-surface glycoprotein receptor expressed in a large number of mammalian cell types. HA binding to CD44 elicits several biomolecular processes, namely inhibition of interleukin (IL)-1β expression, reduction of a disintegrin and metalloproteinase with thrombospondin motifs (ADAMTs) expression, decrease of prostaglandin E2 (PGE2) synthesis, overexpression of the anti-apoptotic heat shock protein 70 (Hsp70), and stimulation of the intrinsic production of proteoglycans inside the joint.

Inhibition of IL-1β expression, a dominant catabolic cytokine present in the inflamed articular joints (12), results in an overall anti-inflammatory effect (7, 13). This inhibition is carried out through induction of mitogen-activated protein kinase phosphatase (MKP)-1, a negative regulator of IL-1β (7, 14). Likewise, IL-1β suppression leads to a decline in the activation of matrix metalloproteinases (MMPs) -1, 2, 3, 9, and 13 (7, 15, 16), thereby decreasing catabolic enzyme activity within the joint cartilage, as well as chondrocyte apoptosis (7, 17–19). Anti-inflammatory effects are further accomplished by a decreased release of other pro-inflammatory mediators, such as IL-6 (7, 20), as well as HA binding to ICAM-1 and subsequent downregulation of the nuclear factor kappa-light-chain enhancer of activated B cells (12). Given that MMPs and IL-6 are linked to inflammatory bone resorption, this HA effect could be bone sparing (7, 20). Most studies on HA mechanisms were done *in vitro*, but reduced levels of peripheral CD4^+^ T lymphocytes, particularly those of the T helper-17 subtype, were shown following intra-articular injection of an HA preparation, suggesting *in vivo* modulation of the inflammatory response by HA with a systemic relevance (21).

Coupling of HA to CD44, *via* reduction of ADAMTs expression (7), a family of peptidases that are involved in the cleavage of important synovial components (including aggrecan, versican, and brevican) (7, 22, 23), enhances chondroprotection and decreases inflammation also through inhibiting PGE2 synthesis and increasing the anti-apoptotic Hsp70 overexpression (7).

Hyaluronic acid–CD44 interaction, in addition with HA binding to ICAM-1, appears to stimulate the intrinsic production of proteoglycans inside the joint (7, 24), apparently depending, at least partially, on the stimulation of insulin-like growth factor-1 pathway (7, 24), thus representing an anabolic activity of HA that may protect the osteoarthritic joint.

### The Role of RHAMM

Hyaluronic acid binding to RHAMM (a cytosolic and surface protein) promotes wound repair, activates pro-migration and invasion functions, regulates cellular responses to growth factors, and plays a role in fibroblast migration and motility (7). This HA activity does also aid in chondroprotection in addition to CD44 binding (7).

### Other Molecular Pathways

Hyaluronic acid has been demonstrated to suppress numerous inflammatory mediators, not only by interacting with CD44 and ICAM-1 but also through toll-like receptors (TLRs) 2 and 4 binding, this way suppressing tumor necrosis factor-α, IL-1-β, IL-17, MMP-13, and inducible nitrogen oxide synthase expression (7, 25).

Hyaluronic acid analgesic effects have been shown to occur at mechano-sensitive stretch-activated ion channels inside the joint, where channel activity is significantly decreased upon HA binding (7).

Hyaluronic acid may suppress fibrinolytic activity mediated by the urokinase-type plasminogen activator and its receptor, this way downregulating the activation of proteinases, including gelatinases and other MMPs (12).

## Structural Properties of the Polysaccharides Used in VS

As shown above, HA dynamic interactions with other molecules modulate cell proliferation and gene expression. It has been suggested that these physicochemical and biologic properties of HA may be due to characteristics such as its MW and the gel formulation of the compound. Most reports seem to agree that HMW HA is superior to low-molecular weight (LMW) HA formulations in achieving the pharmacological effects mentioned. Besides, the gel formulation, as opposed to a saline-soluble preparation, apparently mimics the structural characteristics, particularly the intrinsic viscosity, of the synovial fluid. Allegedly, exogenous HA preparations should be viscous mimicking the “shock absorbing” effect provided by endogenous HA. However, we believe that there are no studies to prove these assumptions (8).

Table 1 summarizes the reviewed studies addressing molecular properties and “viscosupplements” proposed effect.

**Table 1:** Studies discussing molecular properties and viscosupplements efficacy.

Reference	Species/study	Molecular properties studied	Main conclusions
Gomis et al. (26)	Rat/*in vivo*	Hyaluronic acid (HA) elastic and viscous properties	High elastoviscosity impacts analgesia
Homandberg et al. (27)	Bovine/*in vitro*	HA MW	High-molecular weight (HMW) better than low-molecular weight (LMW) in promoting cartilage repair
Sasaki et al. (13)	Human/*in vitro*	HA concentration	HA regulates mRNA expression and protein production even at low concentrations
Waddell et al. (28)	Human/*in vitro*	HA MW	MW irrelevant to the inhibitory effect of matrix metalloproteinase (MMP) activity
Castro et al. (8)	Rat/*in vivo*	Structural properties and rheology of a polysaccharide	Saline and gel formulations of a polysaccharide are equally analgesic
Karna et al. (16)	Human/*in vitro*	HA concentration	Higher concentration increases collagen biosynthesis more than a lower concentration of HA
Yatabe et al. (24)	Human/*in vitro*	HA MW	2,700 kDa HA better than 800 kDa in suppressing aggrecan-degrading a disintegrin and metalloproteinase with thrombospondin motifs species
Peng et al. (17)	Rat/*in vitro*	HA concentration	HA causes apoptosis and dedifferentiation of chondrocytes in a dose-dependent manner
Campo et al. (25)	Mouse/*in vivo*	HA MW	HMW better than LMW in reducing inflammation
Julovi et al. (15)	Human/*in vitro*	HA concentration	Under physiologic concentration of HA produces no effect on MMPs expression
Elmorsy et al. (1)	Rabbit/*in vivo*	HA MW and cross-linking	HMW cross-linked better than LMW exerting chondroprotective effect
Ravera et al. (2)	*In vitro*	Linear vs. cross-linked HA	Cross-linking affects physicochemical properties
Castro et al. (5)	Rat/*in vivo*	Sulfation/oxidation of a polysaccharide alters biologic activity	Structure of the polysaccharide but not viscosity accounts for analgesia

### HMW vs. LMW HA

One reason for HMW HA preparations being superior could be a more effective binding to the CD44 receptor (7). Additionally, HMW HA would produce superior friction coefficients, thus reducing attrition between structures, as compared to LMW HA (29). However, one must consider that *in vitro* evaluations of friction coefficients and intrinsic viscosity may not reflect *in vivo* conditions.

Hyaluronic acid with HMW are increasingly being used in clinical practice such as Hylan G-F 20™—a mixture of a HMW HA with a cross-linked HA preparation, the combination having an MW range of 6 × 10^6^ to 23 × 10^6^ Da (9). However, other HA preparations currently used to treat OA have MW in the order of 1 × 10^6^ Da or less, and most are reported to be effective as well (9). A recent network meta-analysis showed that HA preparations had the highest effect size for analgesia in knee OAs without specifically addressing the MW issue (10).

Meanwhile, studies using large animal models of OA have shown that HA with MW within the range of 0.5 × 10^6^ to 1.0 × 10^6^ Da were generally more effective in reducing indices of synovial inflammation and restoring the rheological properties of synovial fluid than HA with MW > 2.3 × 10^6^ Da (9). It is possible but as yet unproven, that HA preparations within a specific size range could provoke a pattern of CD44 clustering and cross-linking on binding, which then triggers an intracellular signal, whereas the larger HA molecules may occupy these multiple CD44 linking sites but preventing receptor cross-link and a cellular reaction (9). On the other hand, LMW HA preparations may have a greater penetration through the extracellular matrix of the synovium, thereby maximizing its concentration and facilitating interaction with target synovial cells (9). Nevertheless, the interpretation of these studies that favor the use of LMW HA formulations is clouded by the number of protocol variations used (9).

Hyaluronic acid preparations, at least those present in clinical practice, probably do not last more than 3 days inside a joint. Realistically, considering the high synovial blood flow, the effect of movement increasing clearance of intra-articular fluid substances and the presence of proteases that may digest exogenous HA, particularly in inflamed joints, relevant levels of “viscosupplements” probably do not last more than 24 h inside an osteoarthritic knee (29). In keeping with this statement, there are scant data to support any difference in the clinical results obtained with preparations of variable MW (10). Using human synovial explants, Waddell et al. (28) showed that inhibition of MMP activity was similar whether incubating with 1.2 × 10^6^Da HA (Supartz^®^) or 12.8 × 10^6^Da HA (Synvisc^®^) preparations, thus suggesting that mechanisms other than the MW of the VS agents are relevant to their effect. Also, Ronchetti et al. (30) showed HMW hylans (around 10^6^Da) had similar effectiveness as compared to other compounds of LMW (5–7.5 × 10^5^ Da) in providing pain relief in OA (8).

Ghosh and Guidolin (9) have suggested that the ideal MW of HA is still to be determined but it is probably not a fixed size range, varying with characteristics as the extent of inflammation that would affect synovial tissue permeability and cell populations entering the joint, synovial fluid volume, and its rate of clearance.

### Colloidal State

The gel formulation is also claimed to be fundamental for VS effectiveness, mostly considering its analgesic actions. In fact, one of the proposed mechanisms for the antinociceptive activity of hylans is a decrease of the activation of mechano-sensitive receptors located in periarticular structures, as well as in the synovium, by embedding these nerve endings in a highly viscous medium reducing their firing (8, 26). However, this mechanism has been put into scrutiny, as aforementioned (8). As a matter of fact, by using an experimental OA model in rats, it was demonstrated (8) that the intra-articular injection of a protein-free GG derivative—a galactomannan polysaccharide derived from the seed endosperm of the plant *Cyamopsis tetragonolobus—*provided analgesia similar to that of Hylan G-F 20™. Moreover, that GG-derived galactomannan analgesia occurred both with a highly viscous and a saline-soluble preparation, suggesting that the effect was independent of the gel state. Recently, the same group showed (5) that modifications of the chemical structure of that galactomannan, namely oxidation or sulfation, abrogated the analgesia provided by the original compound, using the ACLT rat OA model. Additionally, weekly administrations, starting 14 days until 70 days post-ACLT prevented structural joint damage to the knee, as compared to controls. As a whole, these data suggested that the analgesia provided by a GG-derived galactomannan depends on the chemical structure of the compound, which was also shown to be chondroprotective, questioning that the rheological properties of polysaccharides are responsible for the therapeutic benefit of “viscosupplements” (5).

## Discussion/Conclusion

By reviewing the current literature, it seems that there are multiple mechanisms of action that may explain VS clinical benefits. The dynamic interaction between HA and various cellular receptors appears to be crucial to that therapy, perhaps with a major relevance to CD44 receptor coupling, resulting in several biologic effects such as inhibition of pro-inflammatory mediators and suppression of MMPs activity. Other possible pathways include modulation by ICAM-1, RHAMM, and coupling to TLR-2 and 4, as well as interference with specific ion channels relevant to pain mechanisms inside the joint.

According to some reports, the therapeutic effectiveness of hylans have been claimed to be directly associated with their viscoelastic properties, mostly HMW and gel formulation. However, there may be more than rheology to explain the yet to be proven efficacy of “viscosupplements.” In fact, HA that naturally occurs in the joints has an average MW of 6 × 10^6^ Da, but experiments showed that HA of LMW may present the same or greater effectiveness depending on the specific clinical endpoint and animal species studied. On the other hand, even taking into account the lack of a human study, a plant-derived polysaccharide, obtained from a commonly used compound in the cosmetic, food, and pharmaceutical industries, provided analgesia and chondroprotection regardless of the gel state, this way implying that the biochemical structure and pharmacologic mechanisms rather than rheological properties account to explain the clinical outcome.

Given that intra-articular injection of a protein-free, saline-soluble, easily obtained polysaccharide is probably less complex as compared to that of a highly viscous preparation, it would be interesting to see the experimental data obtained with the GG polysaccharide reproduced in patients with OA.

## Author Contributions

All authors wrote the main manuscript text and approved the final version.

## Conflict of Interest Statement

The authors declare that the research was conducted in the absence of any commercial or financial relationships that could be construed as a potential conflict of interest.

## Abbreviations

ACLT, anterior cruciate ligament transection; ADAMTs, a disintegrin and metalloproteinase with thrombospondin motifs; CD, cluster of differentiation; GG, guar gum; HA, hyaluronic acid; HMW, high-molecular weight; Hsp70, heat shock protein 70; IA-HA, intra-articular hyaluronic acid; ICAM, intercellular adhesion molecule; IL, interleukin; LMW, low-molecular weight; MMP, matrix metalloproteinase; MW, molecular weight; OA, osteoarthritis; PGE2, prostaglandin E2; RHAMM, receptor for hyaluronan-mediated motility; TLRs, toll-like receptors; VS, viscosupplementation.
